# How much cross-sectional area is needed to estimate vessel traits in tropical tree branches?

**DOI:** 10.1093/aob/mcag171

**Published:** 2026-06-17

**Authors:** Robert Muscarella, Julia Valentim Tavares, Carina de Araújo, Silvia Bibbo, Samuel Farrar, Kasia Ziemińska

**Affiliations:** Department of Ecology and Genetics, Uppsala University, Uppsala 752 36, Sweden; Department of Ecology and Genetics, Uppsala University, Uppsala 752 36, Sweden; School of Geography, University of Leeds, Leeds LS2 9JT, UK; Department of Ecology and Genetics, Uppsala University, Uppsala 752 36, Sweden; Department of Plant Biology, Institute of Biology, University of Campinas – UNICAMP, Campinas, CEP 13083-862, Brazil; Department of Ecology and Genetics, Uppsala University, Uppsala 752 36, Sweden; Division of Wood Science and Technology, SLU, Uppsala 756 51, Sweden; Department of Ecology and Genetics, Uppsala University, Uppsala 752 36, Sweden; Tree Anatomy and Function Laboratory, Bielsko-Biała 43-300, Poland

**Keywords:** Quantitative wood anatomy, vessel size, vessel density, vessel grouping, Puerto Rico

## Abstract

**Background and Aims:**

Wood anatomical traits provide key insights into plant physiology, ecology and evolution but their quantification is time-consuming. As a result, measurements are often based on small sample areas or few features, potentially reducing the accuracy and repeatability of estimates. A better understanding of how sampling intensity influences estimation of anatomical traits would improve the efficiency and reliability of wood anatomical studies.

**Methods:**

We annotated all vessels in 164 branch cross-sections from 19 tree species in Puerto Rico and used these to compute the ‘true value’ of seven vessel traits. We then applied a subsampling approach to quantify how error of estimated trait values depended on the proportional and absolute area of the sample annotated. We used variance partitioning to assess how the contributions of within-sample, within-species and among-species variation change with area sampled. We also assessed how sampling intensity affected the number of statistically significant comparisons between species.

**Key Results:**

At low sampling intensity (i.e. ≤10 % of the total cross-section area), absolute percentage differences of estimates from true values ranged from 6 to 18 %, depending on the trait. Across samples, error declined more strongly as a function of proportional area than absolute area sampled. Nonetheless, among-species and among-individual differences explained the majority of variation in the dataset even at low sampling intensity, while variation among replicate subsamples contributed little to the total variation. Depending on the trait, the proportion of statistically significant differences between species increased by 10–21 % from low to high sampling intensity.

**Conclusions:**

Our results suggest that for broad comparisons across species, relatively low sampling intensity can yield robust estimates. However, more sampling may be required for questions demanding greater statistical power, such as within-species or within-individual comparisons. Generally, the design of wood anatomy studies should explicitly consider the accuracy and precision required by the research objectives.

## INTRODUCTION

Wood anatomical traits can provide key insight into plant ecology, physiology and evolution, including the responses of ecosystems to environmental change (Carlquist, 1977, 2001; Fonti *et al*., 2010; Lens *et al*., 2022; Ziemińska *et al*., 2023). The characteristics of water-conducting elements (vessels and tracheids) are of particular interest from an ecophysiological perspective given their role in plant–water relations (Hacke and Sperry, 2001; Tyree and Zimmermann, 2002; Tng *et al*., 2018). Nonetheless, the time-consuming nature of studying microscopic wood anatomy can limit the size and number of samples analysed, which may reduce statistical power and affect conclusions (von Arx *et al*., 2016). In fact, most research in comparative wood anatomy is based on features quantified in relatively small and arbitrarily chosen areas (e.g. <2 mm^2^ area of a larger cross-section) (e.g. Ziemińska *et al*., 2013) or a specified number of features (e.g. 50 elements in total) (e.g. Carlquist, 1977; Scholz *et al*., 2013; Romero *et al*., 2023; Wang *et al*., 2025). A better understanding of how sampling intensity influences the estimation of anatomical traits would improve research efficiency and the reliability of results.

A few studies have investigated how the amount of data from a sample relates to the accuracy and precision of estimated values of wood anatomical traits. For example, Van den Oever *et al*. (1981) tested how the number of measurements affected the precision of estimates for two traits: number of bars per perforation and vessel member length. They concluded that variation in mean trait values and standard deviation was minimal after 25 measurements. The IAWA Committee (1989) recommended a similar threshold, which has since been used in several works (e.g. Carlquist and Hoekman, 1985; Olson and Rosell, 2013; Dutra *et al*., 2023). Meanwhile, Scholz *et al*. (2013) recommended a minimum of 100 vessels for most vessel traits without explicit rationale. Several studies in the context of dendrochronology have examined how sample effort is related to the precision of estimated trait values, and the reliability of vessel chronologies and climate-growth relationships (García-González and Fonti, 2008; Arbellay *et al*., 2012; Seo *et al*., 2014; Diaconu *et al*., 2017; Jourdain *et al*., 2025). A main takeaway from these studies is that optimal sampling intensity is likely to differ for different traits. While these studies collectively provide some insight into the overall question of how sample area or number of measurements relate to wood anatomical features, we have very limited data on cross-species analyses, and especially for diverse groups of species such as tropical angiosperms, as well as for relatively small branches, rather than mature trunk wood.

Some guidelines exist in terms of sample size for vessel and tracheid traits. Scholz *et al*. (2013), for example, mention several of these – mostly based on recommendations for the minimum sample size of vessel elements – but justification for the particular sample sizes mentioned is unclear. As Scholz *et al*. (2013) recognize, sample intensity should ultimately depend on the anatomical variation in a sample and the precision required for the research question, but overall variation is, of course, difficult to assess without a comprehensive assessment. Relatively new and emerging tools for image acquisition and processing are facilitating a shift towards ‘quantitative wood anatomy’, whereby larger areas and numbers of features can be analysed in less time than with traditional methods (von Arx *et al*., 2016; Hwang and Sugiyama, 2021; He *et al*., 2024). While these tools have made valuable advances, they still remain labour-intensive, often still requiring manual editing.

In this study, we built a dataset of 164 branch cross-sections from 19 Puerto Rican tree species. Using a digital slide scanner, we imaged each entire cross-section and then annotated all vessels using a semi-automated procedure. We computed a suite of anatomical traits based on the full cross-section (i.e. the ‘true’ value) and then used a digital subsampling approach to determine how the accuracy and precision of estimates varied with the proportion of the total area or absolute area sampled (i.e. the ‘sampling intensity’). We used variance partitioning to explore how the relative amount of among-species, within-species and within-sample (i.e. replicate subsamples) variation depended on sampling intensity. We asked the following specific questions:

1. How does sampling intensity (i.e. the proportional area of a cross-section annotated) affect the accuracy and precision of estimated values for vessel traits?
2. Does sampling error in vessel trait estimates decrease more strongly with increasing proportional or absolute cross-section area?
3. How does the proportion of variation in estimated trait values attributed to among-species, within-species and residual components change with sampling intensity?
4. Does increased sampling intensity reveal a higher number of statistically significant comparisons between species?

## METHODS

### Study area and species, and sample collection

We conducted this study using wood samples from the Caribbean island of Puerto Rico. We collected samples at six locations across the island, spanning a gradient of mean annual precipitation of ∼1000–3500 mm (Table 1; Daly *et al*., 2003). We focused on 19 abundant tree species that occurred at multiple sampling sites (Table 2). For this study, we measured one cross-section per tree for a total of 164 individuals across the 19 study species. Selection of individual trees for sampling in the field was based on our ability to reach branches with fully matured, sun-exposed leaves. For each focal tree, we collected a canopy branch with sun-exposed leaves using a pole-trimmer. We cut a 7- to 10-cm section of wood ∼70 cm from the distal end of the branch and stored samples in 70 % ethanol.

**Table 1.** Names, locations, mean annual precipitation and sample sizes from the study sites.

Site	Latitude (°N)	Longitude (°W)	Mean annual precipitation (mm)	No. of species	No. of samples
El Verde	18.3333	65.8167	3570	13	77
Cambalache	18.4524	66.5970	1660	6	18
Guajataca	18.36523	66.9705	1900	4	16
Guilarte	18.10968	66.74767	2170	11	38
Guanica	17.9714	66.8687	1040	6	15

**Table 2.** List of study species, botanical family and number of individual cross-sections included in this study.

Species	Family	*N*	No. of sites
*Alchornea latifolia*	Euphorbiaceae	10	2
*Bursera simaruba*	Burseraceae	14	3
*Casearia arborea*	Salicaceae	9	2
*Cecropia schreberiana*	Urticaceae	2	1
*Coccoloba diversifolia*	Polygonaceae	10	2
*Cordia borinquensis*	Boraginaceae	7	1
*Cyrilla racemiflora*	Cyrillaceae	7	1
*Dacryodes excelsa*	Burseraceae	14	2
*Drypetes glauca*	Putranjivaceae	9	2
*Faramea occidentalis*	Rubiaceae	9	2
*Guarea guidonia*	Meliaceae	4	1
*Inga laurina*	Fabaceae	10	2
*Krugiodendron ferreum*	Rhamnaceae	1	1
*Micropholis guyanensis*	Sapotaceae	11	2
*Ocotea leucoxylon*	Lauraceae	14	3
*Poitea florida*	Fabaceae	4	2
*Schefflera morototoni*	Araliaceae	2	1
*Sloanea berteriana*	Elaeocarpaceae	13	3
*Tabebuia heterophylla*	Bignoniaceae	14	3

### Wood sample preparation and scanning

Methods for wood sample preparation and processing follow those in Ziemińska *et al*. (2023). Briefly, we used a WSL sledge microtome (www.wsl.ch/en/services-produkte/microtomes/) to cut a 10- to 30-μm thick transverse section of each sample. We submerged sections for 10 min in an aqueous dye solution of 1 % Safranin O and 1 % Astra blue (Gärtner and Schweingruber, 2013), and then rinsed sections in deionized H_2_O before successively dehydrating them in baths of ethanol of increasing concentration (50, 70, 100, 100, 100 %) for 10 min each. We then mounted sections on glass slides with Euparal and dried them at 60 °C for several days.

### Imaging, annotations and vessel traits

We used a high-resolution slide scanner (Zeiss Axioscan Z.1 widefield microscope system) at the Uppsala University BioVis facility to scan each section for digital analysis of anatomical features. After image capture, we used a semi-automated approach in Qupath (Bankhead *et al*., 2017) to digitally annotate all vessels in each image. Specifically, we first used Qupath to automatically identify features based on colour, size and roundness criteria that we manually tuned for each species. After running this auto-detection step for each sample, we manually cleaned and verified vessel annotations in Qupath, and finally exported the final vessel annotations (as well as annotations delineating the pith and cambium) as vector graphic files (.svg) for further analysis. We used the R packages ‘rsvg’ v.2.7 (Ooms, 2025), ‘grImport2’ v.0.3 (Potter and Murrell, 2024) and ‘sf’ v.1.1 (Pebesma, 2018), as well as functions available in an R package, ‘qwar’ v.0.0.7 (https://github.com/bobmuscarella/qwar), to import annotations and for downstream analyses.

We focus on a suite of seven vessel traits (Table 3) including vessel density, median vessel diameter, hydraulic diameter and theoretical hydraulic conductivity (*K*_h_) of vessels per μm^2^ of sample area. For each sample, we computed *K*_h_ as the sum across all vessels of π*D*^4^/128η, where *D* is vessel diameter (μm) and η is the viscosity index of water (1.002 × 10^−9^ MPa.s at 20 °C), divided by the total sample area excluding the pith (Scholz *et al*., 2013). We computed vessel lumen fraction as the total vessel lumen area divided by total cross-section area excluding the pith. Finally, we computed two indices characterizing the vessel network: the vessel grouping index (VGI, i.e. the total number of vessels divided by the total number of vessel groups) and the solitary vessel index (SVI; the number of solitary vessels divided by the total number of vessel groups) (Scholz *et al*., 2013; von Arx *et al*., 2013). To delineate vessel groups, we first measured the double wall thickness between two adjacent vessels on ≥70 vessel pairs per species. The species-mean values were then used as thresholds to detect vessel groups. With this approach, vessels are categorized as a group if their double wall thickness was equal to or smaller than the threshold (von Arx *et al*., 2013). The R function for identifying vessel groups ‘ves_group_indices’ in the ‘qwar’ package uses the nearest neighbour functionality of the ‘spdep’ v.1.4 R package (Bivand, 2022).

**Table 3.** Vessel traits quantified in this study.

Trait	Abbreviation	Units	Description
Vessel density	V dens	mm^−2^	Average number of vessels per unit area in the sample (Zanne *et al*., 2010)
Median vessel area	V area	μm^2^	Median value of lumen area of vessels in the sample
Hydraulic diameter	*D*_h_	μm	Mean diameter based on the equivalent circle diameter and the Hagen–Poiseuille law (Tyree and Zimmermann, 2002; Scholz *et al*., 2013)
*K*_h_	*K*_h_	µm^4^ s^−1^ MPa^−1^ µm^−2^	Sum of theoretical hydraulic conductivity of vessels in the sample expressed on a per area basis (Scholz *et al*., 2013)
Vessel lumen fraction	V frac	%	Percentage of total sample area (excluding pith) categorized as vessel lumen (Ziemińska *et al*., 2020)
Vessel grouping index	VGI	Unitless	Total of vessels divided by total number of vessel groups (each solitary vessel counts as one group) (Carlquist, 2001; Levionnois *et al*., 2021)
Solitary vessel index	SVI	Unitless	The ratio of solitary vessels to total vessel groups (von Arx *et al*., 2013)

Species-mean values for each anatomical trait are provided in Supplementary Data Table S1.

### Data analysis

**Fig. 1.**
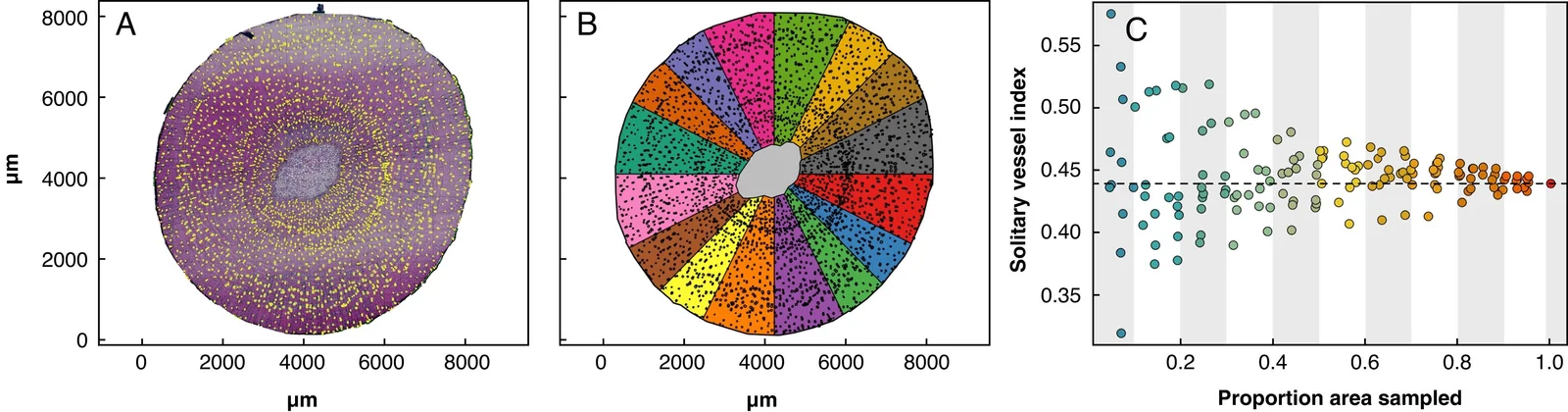
(A) Example of an annotated branch cross-section scan for one sample of *Tabebuia heterophylla* (Bignoniaceae) from the Luquillo Experimental Forest, Puerto Rico. Yellow polygons are annotated vessels. (B) Diagram of the sub-sampling design; each cross-section was digitally divided into 16 equal-angle wedges emerging from the centroid of the section. The grey polygon in the centre is the area manually designated as pith. (C) Values of one example trait (solitary vessel index) for subsample replicates in increasingly large areas of the cross-section based on sampling increasing numbers of contiguous wedges. Point colours and grey shading correspond to bins of sampling intensity; the dashed line represents the ‘true’ trait value for the section (i.e. the value computed based on all vessels in the cross-section). See Supplementary Data Fig. S2 for similar plots summarizing variability of trait estimates across all individuals.

To evaluate the effect of sampling intensity on trait estimates, we first computed the ‘true’ value of each trait for each individual based on the full cross-section (Fig. 1A). We then digitally partitioned each cross-section into 16 wedges radiating from the centroid of the section (Fig. 1B). Note that wedges represent somewhat different areas since the cross-sections are not circular. Subsampling was conducted under two schemes: (1) contiguous wedge sampling and (2) random wedge sampling. For both schemes, sampling intensity was varied by selecting *k* = 1, …, 15 wedges, with ten replicate subsamples per *k*. For contiguous sampling, we randomly selected a set of *k* adjacent wedges defined by proceeding in a clockwise direction. Trait values were calculated for each subsample using all vessels intersecting the selected wedge(s) (Fig. 1C). For random sampling, *k* wedges were randomly selected, without replacement, from the 16 wedges, again with ten replicate subsamples per *k*. Trait values were calculated as above.

To evaluate our first question about how accuracy and precision varied with sampling intensity, iterations were grouped into sampling intensity bins (*b*) defined by 10 % increments of total sapwood area (i.e. 0–10, 10–20 %, etc.). For each replicate, we quantified accuracy as the mean absolute percentage error as:


(1) $$\left|\frac{{\hat{\theta }}_{ibr}-\theta_{i}}{\theta_{i}}\right|$$

where $\theta_{i}$ is the ‘true’ value of the trait for individual *i* and ${\hat{\theta }}_{ibr}$ is the estimated trait value for individual *i*, in sampling intensity bin *b* and replicate *r*. These values were first averaged across replicates within each individual and sampling bin, and then averaged across individuals to obtain the mean percentage error at a given sampling intensity:


(2) $$Error_{b}=\frac{100}{N}\sum_{i=1}^{N}\;\left(\frac{1}{R_{ib}}\sum_{r=1}^{R_{ib}}\;\left|\frac{{\hat{\theta }}_{irb}-\theta_{i}}{\theta_{i}}\right|\right)$$

We quantified precision as the mean within-individual standard deviation of subsample estimates. Specifically, for each individual and sampling bin, the standard deviation of trait estimates across replicates was calculated as:


(3) $$s_{ib}=\sqrt{\frac{1}{R_{ib}-1}\sum_{r=1}^{R_{ib}}\;{\left({\hat{\theta }}_{irb}-{\bar{\hat{\theta }}}_{ib}\right)}^{2}}$$

where ${\bar{\hat{\theta }}}_{ib}$ is the mean estimated trait value for individual *i* in bin *b*. We then averaged these standard deviation values across individuals to obtain the expected variability of estimates at a given sampling intensity:


(4) $$Precision_{b}=\frac{1}{N}\sum_{i=1}^{N}\;s_{ib}$$

To address our second question – whether error in vessel trait estimates declines more strongly with proportional or absolute area sampled – we used linear mixed-effects models to relate sampling error to these two metrics of sampling intensity while accounting for the hierarchical structure of the data. Sampling error was defined as the absolute percentage error (eqn 1). Because replicate subsamples at higher sampling intensities exhibited substantial spatial overlap, we reduced pseudoreplication by retaining a single randomly selected subsample per sampling intensity bin (*b*) for each cross-section. Because error values were strictly positive and right-skewed, we modelled the natural logarithm of absolute percentage error in all analyses.

Models included random intercepts for individual cross-sections and species identity. To allow the relationship between sampling intensity and error to vary among cross-sections, we included random slopes for sampling intensity at the cross-section level. Additionally, we included quadratic terms for sampling intensity because preliminary analyses indicated consistent curvature in the relationship between sampling intensity and error. The full model thus took the form:


$$\begin{array}{rl} log(error_{ij})\;= & \beta_{0}+\beta_{1}log(P_{ij})+\beta_{2}log(P_{ij}{)}^{2}+\beta_{3}log(A_{ij})\\ & \;+\beta_{4}log(A_{ij}{)}^{2}+b_{0i}+b_{1i}log(P_{ij})+b_{2i}log(A_{ij})\\ & \;+b_{s}+\varepsilon_{ij} \end{array}$$

where β_0_ is the expected log(error) at the mean values of proportional and absolute sampling area, P_ij_ and A_ij_ represent the proportional and absolute area sampled, respectively, for subsample bin *j* of cross-section r, and β_1_−β_4_ are fixed-effect coefficients. The term b_0r_ represents a cross-section-specific random intercept, b_1r_ and b_2r_ are random slopes for proportional and absolute area at the cross-section level, b*_s_* is a species-level random intercept and ε*_rj_* is the residual error.

We log-transformed sampling intensity variables prior to analysis to accommodate non-linear (decelerating) declines in error with increasing sampling effort. All predictors were centred and scaled to improve model convergence. For each trait, we used Akaike’s information criterion (AIC) to compare the full model with two reduced models that included either proportional area or absolute area as the sole predictor (each with corresponding quadratic terms and random slopes). Models were fit using the ‘lme4’ package v.2.0 (Bates *et al*., 2013); marginal and conditional *R*^2^ values were calculated using the ‘MuMIn’ package v.1.48 (Bartoń, 2013) to quantify variance explained by fixed effects alone and by both fixed and random effects, respectively.

To answer our third question about the proportion of variation in trait values attributed to among-species, within-species and residual components, we used a nested variance-partitioning approach (Messier *et al*., 2010). Specifically, we used the ‘nlme’ package v.3.1 (Pinheiro *et al*., 2026) to fit linear mixed models with a fixed intercept and nested random effects for species and individual cross-sections. This approach quantified the variation expressed between and within species, as well as residual variance corresponding to variation among replicate subsamples within the 10 % sampling intensity bins described above.

We conducted a final analysis to address our fourth question about how sampling intensity affected inference of species differences. Separately for each trait and sampling intensity bin, we fitted a one-way ANOVA of trait values as a function of species identity. We (1) recorded whether the overall species effect was statistically significant (*P* < 0.05), and (2) quantified the proportion of significant pairwise species comparisons for a given trait based on Tukey’s honest significant difference (HSD) tests, calculated as the fraction of all pairwise comparisons with *P* < 0.05. All analyses were conducted in R v.4.5.2 (R Core Team, 2025).

## RESULTS

**Fig. 2.**
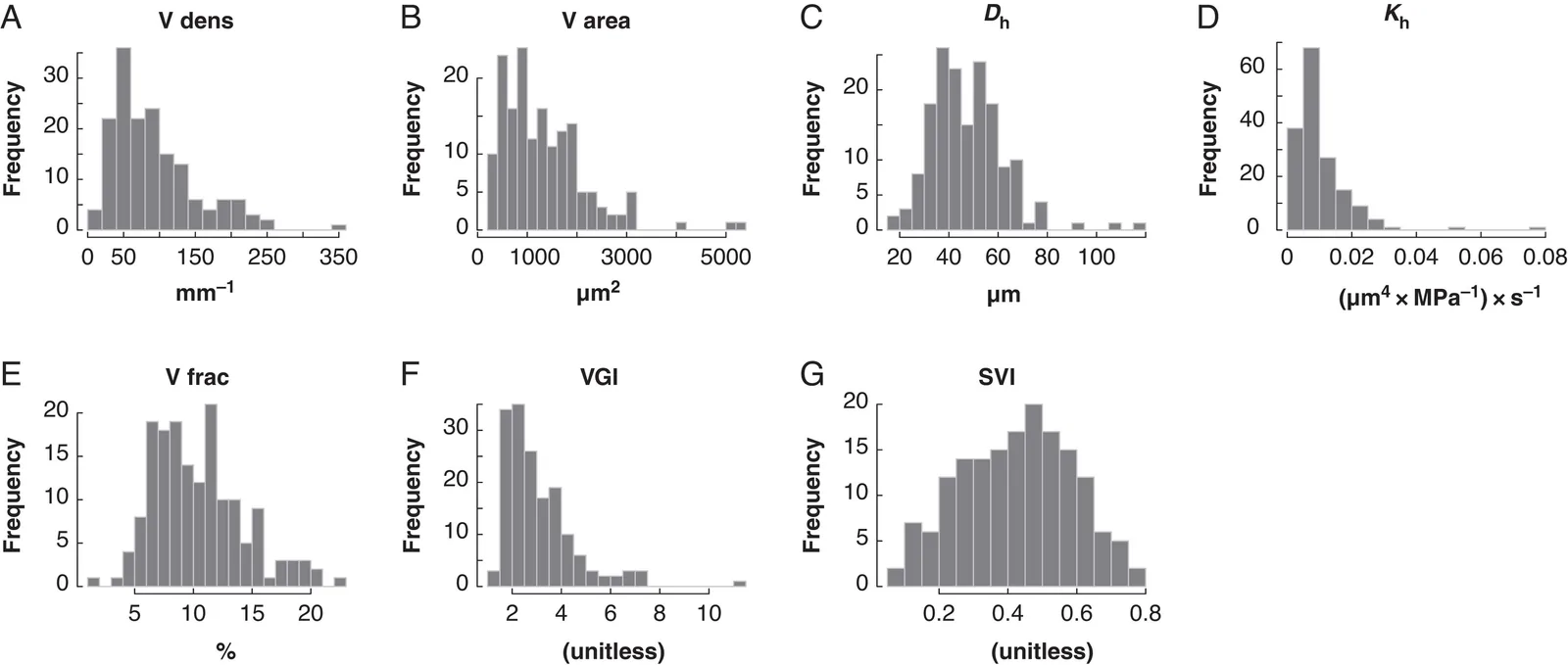
Frequency histograms of the vessel trait data used in this study based on the full sample area and untransformed values for focal individuals. See Table 3 for abbreviations of traits.

The total cross-section area for each of our samples ranged from 8 to 321 mm^2^ (mean ± sd = 69 ± 41 mm^2^). Our full dataset included a broad range of variation across the measured traits (Fig. 2; Supplementary Data Table S1). The relationship between number of vessels and absolute area (mm^2^) with proportional area of the subsamples is shown in Figure S1.

**Fig. 3.**
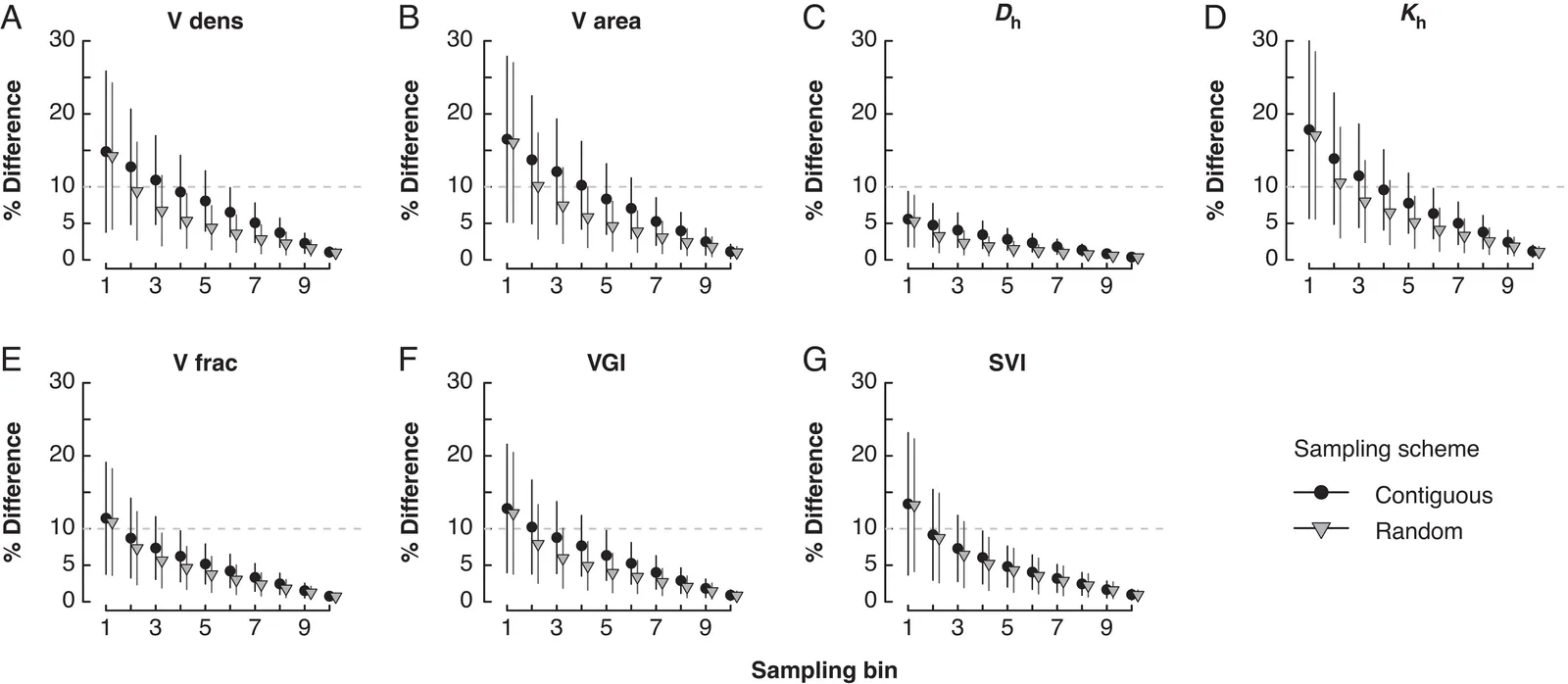
Average absolute percentage difference from the ‘true’ trait value as a function of the binned proportional area sampled from each section. Black circles and grey triangles, respectively, represent the contiguous and random wedge sampling schemes. Error bars reflect the average standard deviation across individuals of replicate estimates in each bin (precision). For comparative purposes, the range of the *y*-axis is consistent across panels; a grey dashed horizontal line at 10 % is included for reference. See Table 3 for abbreviations of traits.

As expected, the accuracy (computed as mean absolute percentage difference from the true trait value) exhibited decelerating declines with increasing sampling intensity (Fig. 3). For contiguous wedge sampling, average error rates at low sampling intensity (i.e. ≤10 % area sampled) varied across traits, ranging from 5.6 to 17.8 %, with *D*_h_ and *K*_h_, respectively, showing the lowest and highest error rates with small sampling intensity. Precision (calculated as the mean standard deviation of replicates within sampling bins) increased with sampling intensity (see error bars in Fig. 3), illustrating lower precision of estimates based on low sampling intensity. Results based on randomly sampled wedges were similar, generally showing slightly lower error rates across traits for intermediate levels of sample intensity (Fig. 3).

For all traits and contiguous wedge sampling, the regression model including proportional area as the only fixed effect received the strongest support based on AIC, when sampling was based on contiguous wedges (Table 4). In other words, after accounting for cross-section-level heterogeneity in sampling responses, absolute area sampled did not typically improve model fit beyond what was explained by proportional area. Results were similar for randomly selected wedges except that the combined models (including both proportional and absolute area) were selected for two traits (VGI and SVI; Supplementary Data Table S2). The selected models explained a substantial fraction of the variation in sampling error; depending on the trait, marginal *R*^2^ ranged from 0.19 to 0.29, indicating that sampling effort explained a large component of variation, and conditional *R*^2^ ranged from 0.48 to 0.62, highlighting additional variation associated with differences among cross-sections and species. Notably, although AIC consistently favoured proportional-area models, absolute-area models yielded slightly higher marginal and conditional *R*^2^ values.

**Table 4.** Summary of mixed effects regression models used to address question 2, based on contiguous wedges (results based on random wedge selection are presented in Supplementary Data Table S2).

Trait	Model	ΔAIC	Marginal *R*^2^	Conditional *R*^2^
Vessel density	Proportion	0.0	0.19	0.48
	Absolute	155.6	0.22	0.59
	Both	13.8	0.20	0.48
Median vessel area	Proportion	0.0	0.28	0.59
	Absolute	178.3	0.32	0.73
	Both	9.1	0.28	0.59
*D*_h_	Proportion	0.0	0.28	0.54
	Absolute	189.9	0.32	0.68
	Both	16.5	0.28	0.54
*K*_h_	Proportion	0.0	0.29	0.53
	Absolute	155.1	0.33	0.67
	Both	17.6	0.29	0.53
Vessel lumen fraction	Proportion	0.0	0.28	0.52
	Absolute	162.3	0.32	0.66
	Both	15.6	0.28	0.52
VGI	Proportion	0.0	0.24	0.60
	Absolute	83.0	0.34	0.66
	Both	1.0	0.29	0.59
SVI	Proportion	0.0	0.25	0.62
	Absolute	107.9	0.32	0.69
	Both	7.3	0.27	0.62

ΔAIC was used to select the best model; marginal and conditional *R*^2^ values respectively quantify the proportion of variation explained by fixed effects only and by fixed + random effects.

**Fig. 4.**
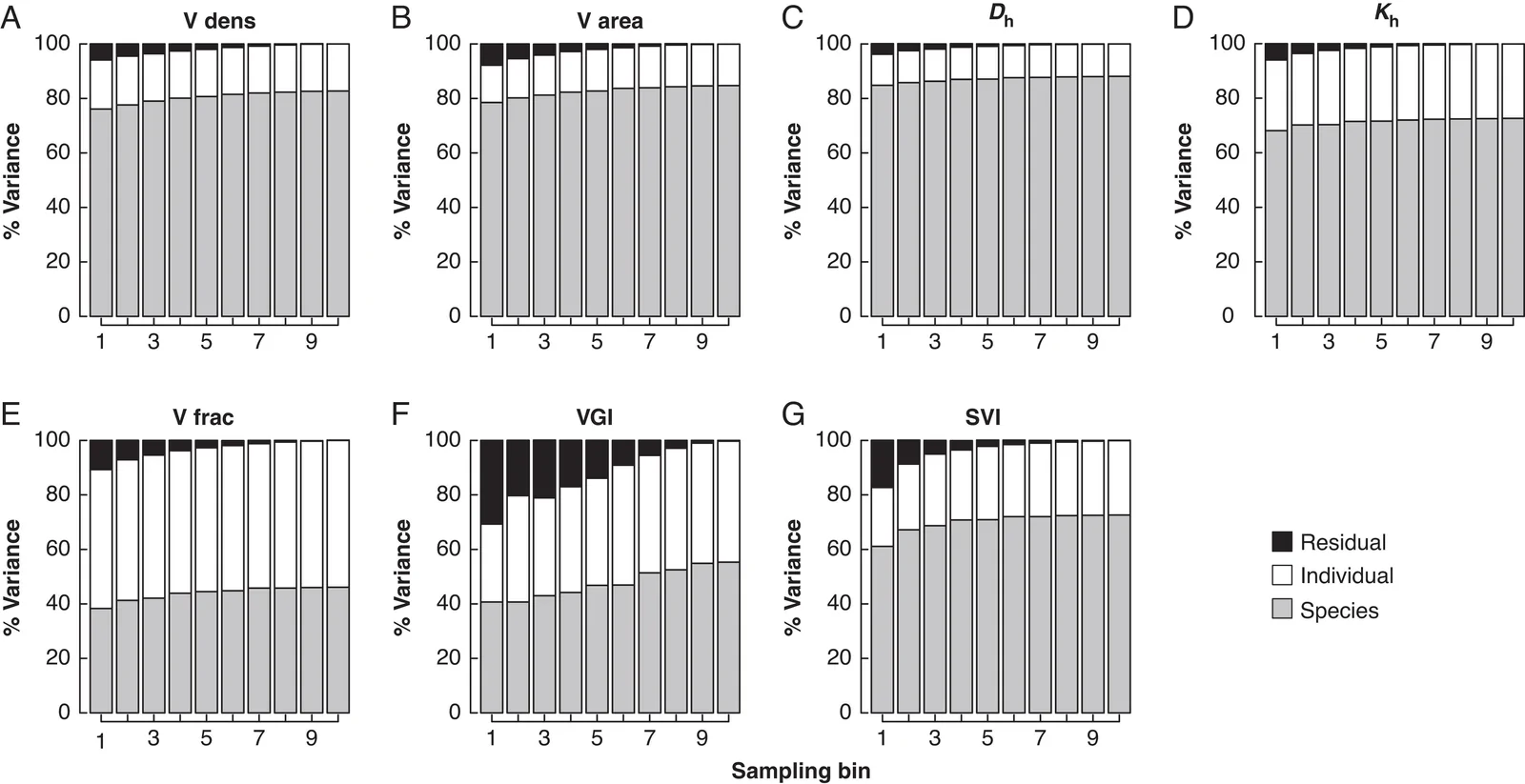
Variance partitioning of vessel traits across 164 samples of 19 Puerto Rican tree species (mean ± sd = 8.7 ± 4.3 individuals per species) based on different proportional areas sampled from the cross-section by sampling contiguous wedges (results based on random wedge sampling are shown in Supplementary Data Fig. S3). All values were log-transformed prior to analysis except VMF and SVI. In each panel, bars from left to right correspond to increasing sampled area bins (e.g. the leftmost bar represents variation based on sampling ≤10 % of the total area). The percentage of variance explained (*y*-axis) by three levels of variation (species, individual and residual) are shown by different colours (see key at top). See Table 3 for abbreviations of traits.

The variance partitioning analysis showed that, for all traits, the majority of variation was explained by differences among species, followed by individuals within species, and then residual variation (subsample replicates within individuals) (Fig. 4). This result was nearly identical for sampling based on random wedges (Supplementary Data Fig. S3). At the lowest sampling intensity (≤10 % area sampled), residual variation accounted for ∼3 to 19 % of the total variation, depending on the trait. The relative proportion explained by residual variation decreased with increasing sampling intensity and, for all traits, was <10 % when at least 30 % of the total section area was analysed.

**Fig. 5.**
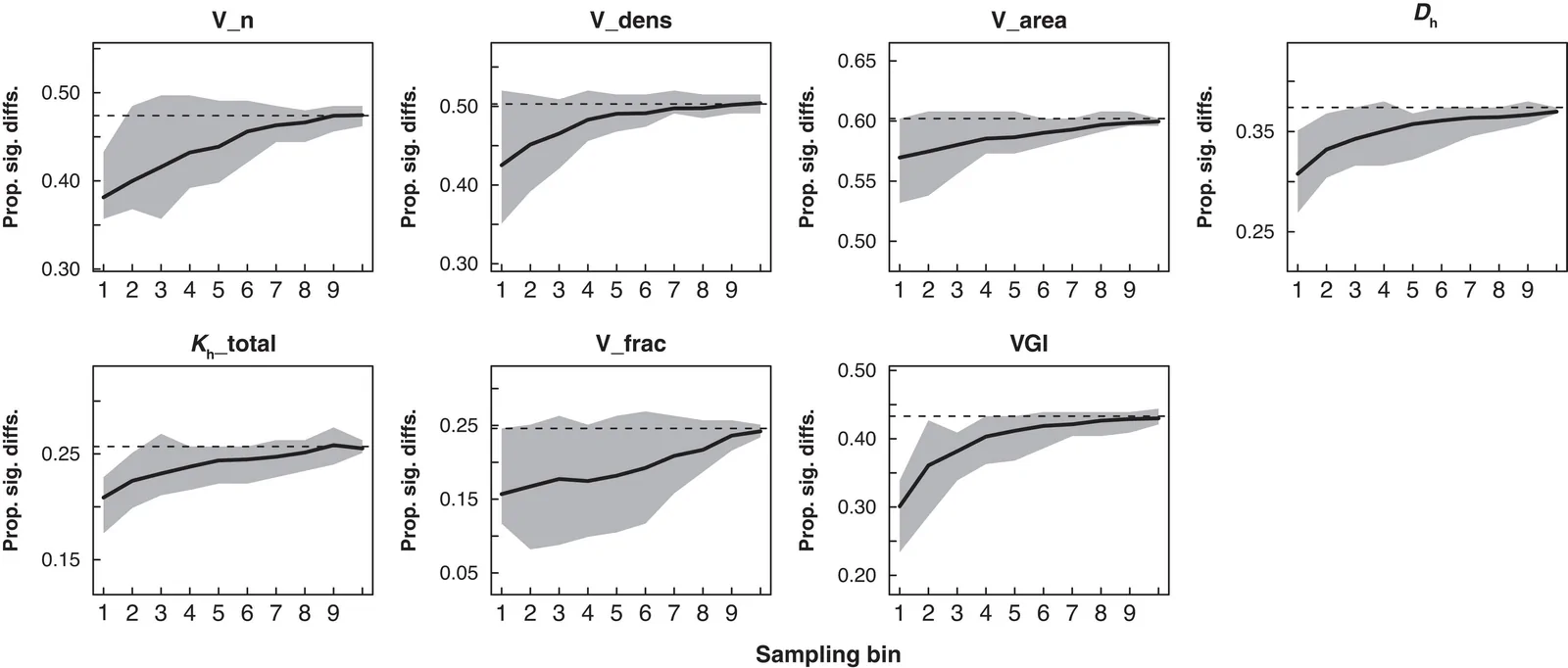
Proportion of pairwise species comparisons with statistically significant differences (*P* < 0.05) when based on different levels of sampling intensity. Solid black lines represent mean values across ten replicates per sampling intensity; grey envelopes represent minimum and maximum values at each sampling intensity. Dashed horizontal lines indicate the proportion of significant pairwise differences when comparisons are based on the entire cross-sections. Each proportion value corresponds to all 171 pairwise comparisons between our 19 study species.

All species-level ANOVA models (i.e. for all traits and at all sample intensities) showed statistically significant overall species differences. The proportion of significant pairwise species comparisons, however, increased with sampling intensity, ranging from a 3 to 13 % increase from the lowest to highest sampling intensities (Fig. 5).

## DISCUSSION

Although new approaches are helping to automate and expedite wood anatomical analyses, careful attention to detail remains crucial for ensuring high-quality results. The time-consuming nature of this work can constrain the number of samples feasible to process in a given study, which can restrict the generalizability of results and limit the statistical power to detect trends. In this study, we used a subsampling approach on a dataset of fully annotated branch cross-sections to examine how the error and precision of estimated vessel traits depended on the area of the sample considered. Our key findings include: (1) the average magnitude of error at low sampling intensity (10 % sample area) varied among traits but declined predictably with increasing sampling intensity, (2) the accuracy of trait estimates was more strongly related to proportional area of a cross-section sampled rather than absolute area, and (3) for our dataset of diverse angiosperms, most variation in vessel traits was explained by among-species variation, even with low sampling intensity (≤10 % of the entire sample annotated). Below we elaborate on these results and discuss them in the context of different types of research questions.

Across the traits we examined, sampling error was highest and most variable at low sampling intensities and declined predictably as larger fractions of the cross-section were annotated. This pattern is consistent with expectations for spatially heterogeneous anatomical features, where limited sampling can lead to biased trait estimates. As sampling intensity increased, estimates increasingly integrated across the heterogeneity within each section, resulting in rapid improvements in accuracy followed by diminishing returns at higher sampling intensities. However, the rate at which error declined differed among traits. *D*_h_, vessel lumen fraction, and vessel grouping indices showed comparatively low error even at low sampling intensities, suggesting that traits reflecting integrated properties of vessel size or spatial organization may be relatively robust to limited sampling.

Tension wood is one of the major sources of circumferential variation in branches. Previous studies have shown that vessel area, density, lumen fraction and grouping indices can differ among tension, opposite and normal wood, although the direction and magnitude of these differences are not always consistent across species (Ruelle *et al*., 2006; Aguayo *et al*., 2014; Ruelle, 2014; Aiso *et al*., 2017; Shashikala *et al*., 2020). Such circumferential heterogeneity could therefore contribute to the higher error observed at low sampling intensities. Nevertheless, the reasons why error differed among traits remain uncertain. One possibility is that some traits are inherently more sensitive to localized variation or extreme values. For example, median vessel area, vessel density and *K*_h_ exhibited relatively high error rates, potentially because these metrics respond strongly to local fluctuations in vessel characteristics. In contrast, *D*_h_ may be less sensitive to circumferential variation because it disproportionately weights larger vessels, thereby reducing the influence of smaller-scale fluctuations in vessel area among sections. Vessel lumen fraction also showed relatively low error. Because lumen fraction reflects the combined effects of vessel size and vessel density, changes in one component may partially offset changes in the other, resulting in greater overall stability. Consistent with this interpretation, vessel lumen fraction tends to vary less across species than vessel area or vessel density (Zanne *et al*., 2010; Chen *et al*., 2025). Vessel grouping indices likewise exhibited relatively low error at low sampling intensities, suggesting either that grouping patterns were comparatively stable around the circumference or that the numerator and denominator of these indices changed proportionally. For example, in VGI, increases in vessel number may be accompanied by proportional increases in the number of vessel groups.

Our mixed-effects models showed that the proportion of the cross-section sampled typically offered a more parsimonious prediction of accuracy of vessel trait estimates than absolute area sampled. Intuitively, this pattern probably arises because proportional area inherently accounts for differences in total cross-section size: a given absolute area may correspond to very different fractions of small versus large sections, whereas proportional coverage scales consistently across individuals. By contrast, a fixed absolute area would capture a smaller fraction of a large cross-section and thus may fail to represent its spatial heterogeneity. Consequently, we argue that the proportional area of a section provides a more reliable predictor of sampling accuracy than absolute area across species and cross-section sizes. The actual amount of difference between the methods (and its relevance for a given study) would depend on the overall size and characteristics of the samples (e.g. amount of within-individual heterogeneity), as well as the research objectives. Practically, we suggest that researchers may benefit from targeting (and reporting) a certain proportional coverage rather than a fixed absolute area (or specific number of elements) when designing sampling protocols for wood anatomy studies. Minimally, these values should be reported. Note that our conclusions apply specifically to branch cross-sections, and similar studies would help assess precision and accuracy for trunk-wood.

For all traits, the majority of variation was explained by among-species differences, followed by within-species and then residual variation, with the last category decreasing as sampling intensity increased. Wood of tropical species is known to be very diverse (Fichtler and Worbes, 2012; Fortunel *et al*., 2014; Ziemińska *et al*., 2015; Dória *et al*., 2022) but reports comparing hierarchical sources of variation across a diverse set of species are scarce. A previous study on ten Puerto Rican tree species from a single site (using a completely different dataset than analysed here) found that among-species differences explained the largest proportion of vessel trait variation (Ziemińska *et al*., 2023). Similarly, Messier *et al*. (2018) observed that for 24 temperate species, among-species variation was larger than within-species variation (explicitly assigned to local microhabitat differences), although for most vessel traits the residual variation was the largest component. These studies analysed a subsample of a stem cross-section (a core from a trunk or a wedge of a small stem cross-section) and they did not report their sampling intensity. Nevertheless, our finding that even low sampling intensity can detect the variance hierarchy quite well, at least across a diverse set of species, supports these previous results. Other previous work focused on within-species comparisons across a broad geographical or climatic range (e.g. Hajek *et al*., 2016; Fajardo *et al*., 2022), extreme climatic events (e.g. drought, Rodríguez-Ramírez *et al*., 2022) or experimentally induced treatments (Zhang *et al*., 2020; e.g. water regimes, nitrogen addition, Plavcová *et al*., 2023). Further tests are needed to determine how our results apply to such study designs.

Our findings are most relevant for comparative studies in ecology, physiology and evolution that focus on functionally relevant vessel traits for understanding hydraulic safety and efficiency. For these types of studies, we show that relatively low sampling intensities are probably adequate when the aim is to assess broadscale variation across diverse sets of species. For example, similar to our results, Messier *et al*. (2018) and Ziemińska *et al*. (2023) found larger interspecific than intraspecific variation in a diverse set of tropical and temperate tree species, despite analysing a relatively small subsample of wood cross-section. Moreover, our analyses of pairwise comparisons show that relatively low sampling intensities (10–20 % of the cross-sectional area) provide fewer statistically significant differences between species. Studies comparing species in terms of vessel traits will have a higher chance to detect significant relationships when they measure larger proportions of wood cross-sectional area. For research objectives that require higher accuracy [e.g. intraspecific studies (e.g. Hajek *et al*., 2016) or wood identification for forensic purposes (e.g. Gasson *et al*., 2010)], a higher sampling intensity or broader range of traits is typically required (i.e. more than just vessel characteristics). In this case, the combination of higher sampling intensity and/or many features may outweigh variability of a given character (IAWA Committee, 1989; Low *et al*., 2022). Additional studies like ours to empirically establish sampling intensity required for other traits could be valuable.

Based on our experience generating the dataset used in this study, the time required for manual editing of vessel annotations varied greatly among samples depending on the size of the sample, the total number of vessels, and the quality of the section and image. Specifically, it took from approximately 2 to 10 h to annotate each section, which corresponds to months of labour for a large-scale comparative dataset such as ours. Reducing the sampling intensity to 25 % would have resulted in approximately 10 % error rates, on average, while maintaining the overall ability to detect species- and community-level variation. Nonetheless, it is worth noting that, in our dataset, the 0–10 % bin of proportional sampling area corresponded to a wide range of absolute area (mean [range] = 4.1 mm^2^ [0.32–25.7 mm^2^]) and number of vessels (mean [range] = 331 [15–1786]), which, for many samples, is still substantially larger than standard recommendations of sample size (e.g. Scholz *et al*., 2013). The strong trade-off between sampling intensity and potential error rates is important to consider for future studies.

In order to capture radial variation across each section, we designed our subsampling approach using wedges that expanded outwards from the center of each section. Even in tropical trees that are less often recognized to produce annual growth rings, radial anatomical variation can be substantial (Drew, 1998; Fichtler and Worbes, 2012; Ziemińska *et al*., 2023). We would expect an even higher degree of uncertainty (i.e. average difference from the ‘true’ trait value) from subsample areas based on sections that do not capture radial variation (e.g. sampling a small square). Generally, subsampled areas of cross-sections should be chosen to be as representative of the full section as possible (García-González and Fonti, 2008).

Our results lead to several concrete recommendations for future studies that characterize variation in vessel traits. First, among the traits considered here, vessel area, density and *K*_h_ had the lowest precision with low sampling intensity whereas *D*_h_, vessel fraction and the grouping indices tended to have higher precision at low sampling intensity. Researchers should consider these differences across traits, as well as the accuracy and precision required for their research objective, when designing the sampling approach. The values reported here for Puerto Rican trees may provide a helpful baseline but further additional work with more species and additional traits would be valuable to refine this assessment. Second, we suggest researchers should clearly report the proportional and absolute area of the sample annotated when reporting trait values. This additional information could help readers assess the potential error associated with the estimate. Moreover, researchers may find value in targeting a given proportional area per sample rather than an absolute area or number of vessels to maintain some consistency in terms of relative sampling intensity across samples in a given study. Finally, to reiterate a suggestion from von Arx *et al*. (2016), care should be taken to prepare and image high-quality wood sections as this will typically pay off many times when it comes to time spent on manual editing and data quality.

## CONCLUSIONS

Our results indicate that sampling a relatively small proportional area (5–10 %) of a cross-section can provide a robust estimate of some vessel trait values, especially if the aim is a broad comparison across species. More intense sampling would probably be required when considering more subtle differences (e.g. within species) or to achieve higher statistical power. The degree to which the research question requires high accuracy and precision for a given trait value should be considered explicitly when designing a methodology for wood anatomy research.

## Supplementary Material

mcag171_Supplementary_Data

## Data Availability

Summary data and code to reproduce analyses are available online at https://github.com/bobmuscarella/Wood-anatomy-sampling. The custom R package qwar is available at https://github.com/bobmuscarella/qwar.
