# Supplementary material for: How much cross-sectional area is needed to estimate vessel traits in tropical tree branches?
Source: Ann Bot. 2026 Jun 17;138(4):971–81. doi: 10.1093/aob/mcag171 (PMC13595280; doi:10.1093/aob/mcag171)
Supplement: Supplementary file 1 [file mcag171_supplementary_data.docx]

**Supporting Materials**

**How much cross-sectional area is needed to estimate vessel traits in tropical tree branches?**

Robert Muscarella^1^*, Julia Valentim Tavares^1,2^, Carina de Araújo^1,3^, Silvia Bibbo^4^, Samuel Farrar^1^, Kasia Zieminska^5^

**Table S1.** Species-level values for vessel traits used in this study.

**Table S2.** Summary of mixed effects regression models used to address question 2, based on randomly selected wedges.

**Figure S1.** Proportional area of cross-section sampled versus the number of vessels included in the sample and the absolute area (mm^2^) of the sample.

**Figure S2.** Estimated values for each trait based on different proportions of the total section area sampled across all individuals.

**Figure S3.** Variance partitioning of vessel traits species based on different proportional areas sampled from the cross-section using random selection of wedges.

**Table S1.** Species-level mean values (± standard deviation) for traits used in this study.

| **Species** | **Absolute area (mm^2^)** | **N vessels** | **Vessel density (mm^-2^)** | **Median vessel lumen area (μm^2^)** | **Dh (μm)** | **Kh total (/1000)** | **Vessel lumen fraction (%)** | **VGI** | **SVI** |
| --- | --- | --- | --- | --- | --- | --- | --- | --- | --- |
| *Alchornea latifolia* | 110.5 ± 62.1 | 2500 ± 612 | 25.6 ± 7 | 2483 ± 1035 | 73.4 ± 9.6 | 2073 ± 1752 | 7.86 ± 1.76 | 2.59 ± 0.44 | 0.51 ± 0.07 |
| *Bursera simaruba* | 67.1 ± 25.8 | 4461 ± 2000 | 67.6 ± 28.4 | 2189 ± 686 | 58.2 ± 8.7 | 1399 ± 760 | 15.03 ± 4.63 | 2.63 ± 0.81 | 0.48 ± 0.09 |
| *Casearia arborea* | 32.6 ± 24.9 | 5055 ± 2595 | 179.7 ± 44.3 | 1057 ± 391 | 39.2 ± 6.8 | 377 ± 372 | 18.06 ± 2.95 | 5.93 ± 2.56 | 0.3 ± 0.06 |
| *Cecropia schreberiana* | 134.2 ± 102.6 | 1932 ± 606 | 17.9 ± 9.2 | 4754 ± 826 | 111.7 ± 7.4 | 7771 ± 4286 | 12.33 ± 4.79 | 1.81 ± 0.06 | 0.59 ± 0.07 |
| *Coccoloba diversifolia* | 59.9 ± 18.6 | 2961 ± 879 | 53 ± 22.1 | 685 ± 241 | 45.3 ± 7.4 | 317 ± 157 | 5.54 ± 1.24 | 4.7 ± 1.37 | 0.2 ± 0.07 |
| *Cordia borinquensis* | 58.9 ± 27.4 | 2145 ± 921 | 37.1 ± 5.5 | 1808 ± 323 | 57.6 ± 5.4 | 632 ± 359 | 7.74 ± 1.24 | 2 ± 0.18 | 0.54 ± 0.05 |
| *Cyrilla racemiflora* | 63.3 ± 22.8 | 12303 ± 3581 | 203.7 ± 41 | 509 ± 92 | 30.9 ± 2.4 | 297 ± 159 | 12.36 ± 2.47 | 4.12 ± 1.52 | 0.49 ± 0.05 |
| *Dacryodes excelsa* | 51.6 ± 13 | 3099 ± 1016 | 59.4 ± 9.2 | 1796 ± 296 | 53.8 ± 4.8 | 647 ± 279 | 11.43 ± 2.24 | 1.8 ± 0.17 | 0.63 ± 0.05 |
| *Drypetes glauca* | 59.7 ± 28.5 | 5576 ± 2616 | 96.9 ± 24 | 820 ± 146 | 38.5 ± 3 | 306 ± 157 | 9 ± 1.67 | 3.22 ± 0.62 | 0.3 ± 0.05 |
| *Faramea occidentalis* | 52.4 ± 31.7 | 8927 ± 3806 | 198.7 ± 75.1 | 345 ± 114 | 24.2 ± 3.9 | 100 ± 101 | 7.15 ± 1.42 | 3.83 ± 1.33 | 0.36 ± 0.1 |
| *Guarea guidonia* | 30.2 ± 14.2 | 3216 ± 1421 | 109.1 ± 16.9 | 588 ± 170 | 33.2 ± 4.2 | 108 ± 87 | 7.49 ± 1.44 | 2.23 ± 0.33 | 0.46 ± 0.04 |
| *Inga laurina* | 71.2 ± 29.2 | 2584 ± 1028 | 37.1 ± 8.1 | 1362 ± 229 | 56 ± 6.4 | 692 ± 488 | 6.78 ± 2.05 | 1.75 ± 0.3 | 0.67 ± 0.07 |
| *Krugiodendron ferreum* | 60.8 ± NA | 12432 ± NA | 204.3 ± NA | 293 ± NA | 24.2 ± NA | 104 ± NA | 7.23 ± NA | 3.25 ± NA | 0.45 ± NA |
| *Micropholis guyanensis* | 58.4 ± 23.7 | 6744 ± 2226 | 122.9 ± 26.5 | 776 ± 264 | 38.1 ± 5 | 382 ± 213 | 10.68 ± 1.91 | 4.27 ± 0.68 | 0.16 ± 0.04 |
| *Ocotea leucoxylon* | 70.4 ± 33.2 | 4477 ± 1394 | 69.7 ± 18.5 | 1629 ± 425 | 50.9 ± 5.6 | 829 ± 560 | 11.67 ± 2.71 | 2.37 ± 0.53 | 0.46 ± 0.15 |
| *Poitea florida* | 39.7 ± 26.6 | 3100 ± 1319 | 94 ± 49.3 | 930 ± 484 | 43.7 ± 8.7 | 322 ± 305 | 9.32 ± 1.86 | 2.92 ± 1.14 | 0.47 ± 0.11 |
| *Schefflera morototoni* | 123.9 ± 42.2 | 5344 ± 1969 | 42.9 ± 1.3 | 2971 ± 199 | 65.4 ± 2.8 | 2483 ± 1299 | 12.76 ± 1.37 | 2.22 ± 0.4 | 0.47 ± 0.11 |
| *Sloanea berteriana* | 65.5 ± 23 | 5259 ± 1109 | 86.5 ± 27.3 | 1037 ± 302 | 44.8 ± 6 | 579 ± 347 | 10.19 ± 1.51 | 2.62 ± 0.57 | 0.44 ± 0.07 |
| *Tabebuia heterophylla* | 50 ± 21 | 5562 ± 2674 | 110 ± 18.4 | 860 ± 270 | 38.9 ± 5.5 | 364 ± 267 | 10.62 ± 3.01 | 3.28 ± 0.74 | 0.33 ± 0.1 |

**Table S2.** Summary of mixed effects regression models used to address question 2, based on randomly selected wedges (results based on adjacent wedge selection is presented in the main text). ΔAIC was used to select the best model; marginal and conditional R^2^ values respectively quantify the proportion of variation explained by fixed effects only and by fixed + random effects.

| **Trait** | **Model** | **ΔAIC** | **Marginal R^2^** | **Conditional R^2^** |
| --- | --- | --- | --- | --- |
| Vessel number | Proportion | 0.0 | 0.24 | 0.35 |
|  | Absolute | 95.1 | 0.26 | 0.43 |
|  | Both | 9.41 | 0.24 | 0.36 |
| Vessel density | Proportion | 0.0 | 0.24 | 0.35 |
|  | Absolute | 95.6 | 0.26 | 0.43 |
|  | Both | 9.41 | 0.24 | 0.36 |
| Median vessel area | Proportion | 0.0 | 0.27 | 0.40 |
|  | Absolute | 128.6 | 0.38 | 0.50 |
|  | Both | 16.9 | 0.27 | 0.40 |
| Dh | Proportion | 0.0 | 0.28 | 0.37 |
|  | Absolute | 123.1 | 0.30 | 0.46 |
|  | Both | 13.7 | 0.28 | 0.37 |
| Kh total | Proportion | 0.0 | 0.31 | 0.33 |
|  | Absolute | 180.7 | 0.31 | 0.43 |
|  | Both | 14.3 | 0.31 | 0.33 |
| Vessel lumen fraction | Proportion | 0.0 | 0.28 | 0.32 |
|  | Absolute | 175.45 | 0.29 | 0.42 |
|  | Both | 17.51 | 0.28 | 0.32 |
| VGI | Proportion | 14.5 | 0.26 | 0.40 |
|  | Absolute | 97.6 | 0.31 | 0.42 |
|  | Both | 0.0 | 0.29 | 0.39 |
| SVI | Proportion | 2.4 | 0.26 | 0.47 |
|  | Absolute | 90.2 | 0.30 | 0.50 |
|  | Both | 0.0 | 0.28 | 0.46 |

**Figure S1.** Proportional area of cross-section sampled versus (A, C) number of vessels included in the sample and (B, D) absolute area (mm^2^) of the sample. Panels A and B correspond to contiguous wedge sampling; panels C and D correspond to randomly sampled wedges. Each line represents one individual tree; lines are based on average values of x- and y-variables based on 10 replicates per sampling intensity bin.

**Figure S2.** Estimated values for each trait based on different proportions of the total section area sampled across all individuals in the study. The trait values are z-transformed (estimate - mean / standard deviation) within each individual cross-section to make them comparable across cross-sections.

**Figure S3**. Variance partitioning of vessel traits across 164 samples of 19 Puerto Rican tree species based on different proportional areas sampled from the cross-section (using random selection of wedges). All values were log-transformed prior to analysis except VMF and SVI. In each panel, bars from left to right correspond to increasing sampled area bins (e.g., the leftmost bar represents variation based on sampling ≤10% of the total area). The percentage of variance explained (y-axis) by three levels of variation (species, individual, and residual) are shown by different colors (see legend at top). See Table 2 in the main text for abbreviations of traits.
